# Effects of surgery on survival of patients aged 75 years or older with oral tongue squamous cell carcinomas

**DOI:** 10.1038/s41598-021-85647-y

**Published:** 2021-03-16

**Authors:** Yujiao Li, Chu Chu, Chaosu Hu

**Affiliations:** 1 Department of Radiation Oncology, Fudan University Shanghai Cancer Center, 270 Dong An Road, Shanghai, 200032 China; 2 Department of Oncology, Shanghai Medical College, Shanghai, China; 3 Department of Radiation Oncology, Sun Yat-Sen University Cancer Center, Guangzhou, Guangdong China

**Keywords:** Head and neck cancer, Oral cancer, Head and neck cancer, Oral cancer

## Abstract

The objective of this study is to assess prognostic value of surgery for elderly oral tongue squamous cell carcinomas (OTSCC) patients. Patients with OTSCC were extracted from the SEER database between 2010 and 2014. The distributions of categorical demographic and clinicopathological characteristics were determined for different age groups: the 75–79, 80–84, and 85–102 years old groups. Univariate and multivariate analyses were performed to determine the effects of each variable on survival. A total of 1064 patients were analyzed. 75–79 years old patients tended to be male and rate of surgery declined with advancing age (P < 0.001). 75–79 years old patients more frequently presented with advanced stage compared to their older peers (P = 0.002). Compared to surgery groups, the hazard ratios for no surgery groups were 2.856 (95% CI 2.267–3.599; (P < 0.001)) for OS and 3.687 (95% CI 2.561–5.308; (P < 0.001)) for CSS in multivariable analysis. In subgroup analysis, the effect of no surgery was significantly associated with a higher risk of poor CSS in patients aged 75–79 years, 80–84 years and 85–102 years (P < 0.001, respectively). Our results showed that there were a series of factors contributing to poor outcomes in the elderly OTSCC patients, including clinicopathological characteristics and surgical management. Surgical resection is significantly associated with an improved OS and CSS, but further exploration in larger prospective clinical trials and better prognostic and predictive tools for select old patients for surgery are needed.

## Introduction

Oral tongue squamous cell carcinoma (OTSCC) is one of the most frequent head and neck cancers, accounting for 20% of these cancers^1–3^. Approximately 24% to 55% of the patients were reported to have locally advanced disease at presentation, and the 5-year overall survival is 15% to 45%^4,5^. The primary therapeutic strategy for patients has been surgery, and systemic therapy has been reserved based on a multitude of factors.

The malignancy is rare below the age of 40, with a mean age at diagnosis of 50–60 years old^1,5–7^. The poor prognosis associated with advanced age was thought to be due to poor immunologic defense against cancer, more aggressive histological type^8^, or less aggressive therapy^9–13^. By the year 2030, 20% of Americans will be older than 65 years, with those aged > 85 years representing the fastest-growing subset^14^. The older population experiences the greatest suffering caused by this cancer; however, elderly patients are greatly underrepresented in the scientific data and age-restrictive exclusion criteria are commonplace in clinical trials^15^. Therefore, the aim of this study is to assess the impact of surgery on long-term overall survival (OS) and cancer-specific survival (CSS) in a large cohort of elderly patients with OTSCC.

## Results

### Clinical characteristics of all patients

A total of 1064 OTSCC patients were included in this population-based study, with a median age of 81 years old (range 75–102 years old). 438 (41.2%), 342 (32.1%), and 284 (26.7%) patients were aged 75–79, 80–84, and 85–102 years old, respectively. Among the cohort of the patients, 65.9% and 34.1% patients were stage I–II and stage III–IV, respectively. Most of OTSCC occurs on the anterior 2/3 of tongue (27.7%). 75–79 years old patients tended to be male and rate of surgery declined with advancing age (P < 0.001, respectively). 75–79 years old patients more frequently presented with advanced stage compared to their older peers (P = 0.002). Only 16.8% (179/1064) didn’t receive surgery to primary tumor. The clinicopathological features stratified by age at diagnosis are listed in Table 1.

**Table 1:** Demographic features of patients stratified by age at diagnosis.

Features	n	%		75–79	80–84		85–102	P
				n	n		n	
**Gender**								0.000
Male	534		50.2	241	180		113	
Female	530		49.8	197	162		171	
**Race**								0.098
Caucasian	930		87.4	373	297		260	
Asian	93		8.7	44	34		15	
African American	41		3.9	21	11		9	
**Stage**								0.002
I–II	701		65.9	263	234	204		
III–IV	363		34.1	175	108	80		
**T classification**								0.327
T1	532		50.0	224	171	137		
T2	338		31.8	125	117	96		
T3	115		10.8	47	33	35		
T4a	76		7.1	40	20	16		
T4b	3		0.3	2	1	0		
**N classification**								0.060
N0	803		75.5	314	260	229		
N1	121		11.4	68	34	19		
N2a	4		0.4	1	1	2		
N2b	107		10.1	44	36	27		
N2c	23		2.2	10	8	5		
N3	6		0.6	1	3	2		
**Grade**								0.306
1	297		27.9	113	94	90		
2	579		54.4	248	180	151		
3	188		17.7	77	68	43		
**Surgery therapy**								0.000
Yes	885		83.2	379	287	219		
No	179		16.8	59	55	65		
**Tumor location**								0.683
Dorsal surface of tongue	53		5.0	23	14	16		
Border of tongue	214		20.1	89	67	58		
Ventral surface of tongue	124		11.7	58	36	30		
Anterior 2/3 of tongue	295		27.7	115	107	73		
Overlapping lesion of tongue	57		5.4	23	14	20		
Tongue anterior, NOS	321		30.2	130	104	87		

*NOS* not otherwise specified.

### Survival

**Figure 1:**
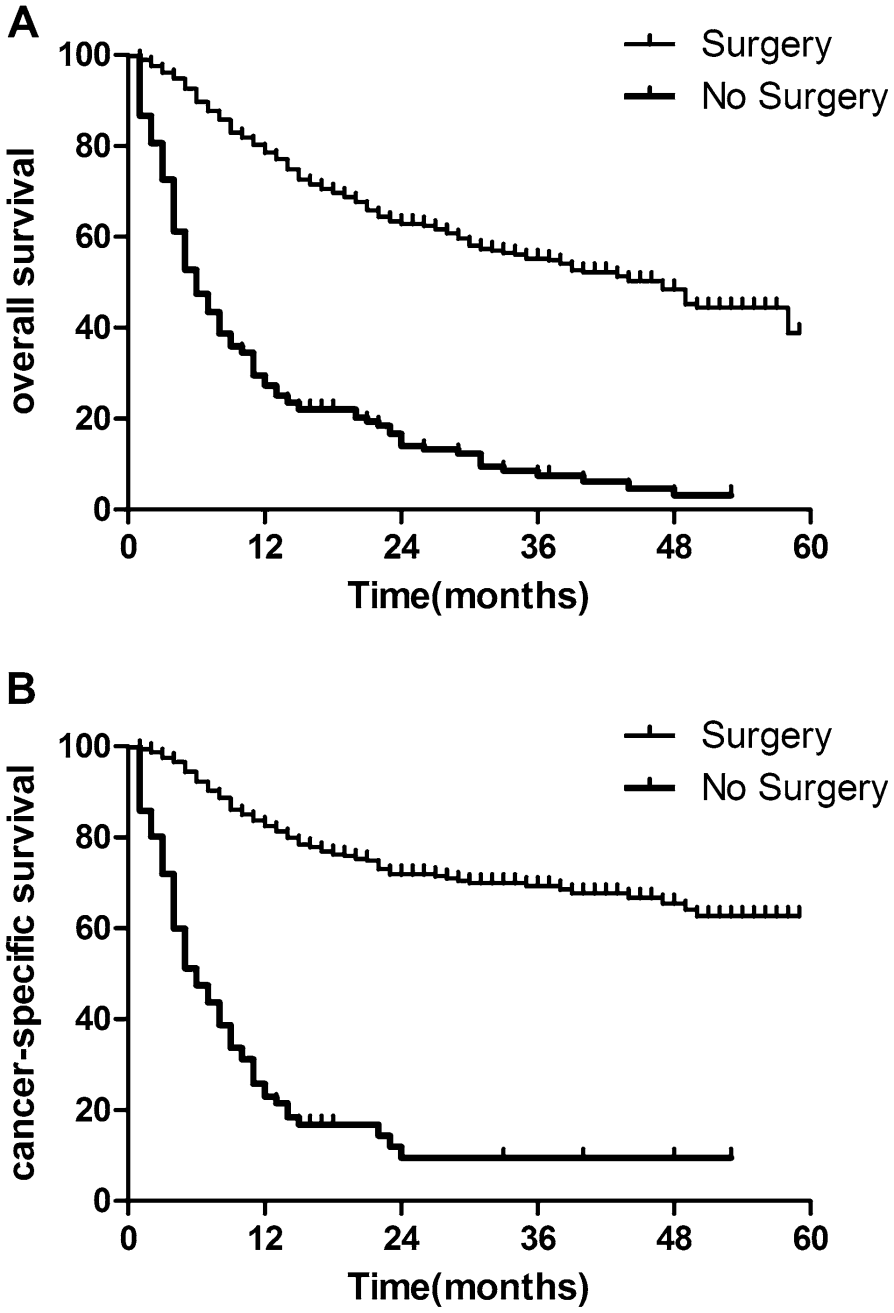
Kaplan–Meier analysis of OS (**A**) and CSS (**B**) in the surgery and no surgery groups.

The median OS was 14.0 months (range 0–59 months). Univariate regression analyses showed that age, gender, grade, tumor location, stage, T category, N category, and surgery therapies to the primary tumor were significant risk factors of overall survival (P < 0.05). Figure 1 illustrated that the surgery groups showed significantly better OS and CSS than the no surgery groups (P < 0.05).

In the multivariable analysis, compared to surgery groups, the hazard ratios for no surgery groups were 2.856 (95% CI 2.267–3.599; (P < 0.001)) for OS and 3.687 (95% CI 2.561–5.308; (P < 0.001)) for CSS, respectively (Table 2).

**Table 2:** Multivariable logistic regression for overall survival and cancer-specific survival in the SEER cohort.

Prognostic factor	Overall survival				Cancer-specific survival			
	P value	HR	Lower 95% CI	Higher 95% CI	P value	HR	Lower 95% CI	Higher 95% CI
**Age**	0.000				0.000			
75–79		1 (reference)				1 (reference)		
80–84		1.263	0.999	1.595		1.347	0.935	1.940
85–102		1.839	1.454	2.325		1.718	1.188	2.484
**Gender**	0.000				0.819			
Male		1 (reference)				1 (reference)		
Female		0.751	0.621	0.908		0.833	0.618	1.124
**T classification**	0.000				0.000			
T1		1 (reference)				1 (reference)		
T2		2.096	1.660	2.647		2.247	1.516	3.331
T3		3.493	2.568	4.750		4.423	2.688	7.280
T4a		2.568	1.791	3.682		2.100	1.175	3.755
T4b		2.222	0.608	8.116		22.305	4.821	103.190
**N classification**	0.000				0.000			
N0		1 (reference)				1 (reference)		
N1		1.072	0.807	1.424		1.197	0.786	1.825
N2a		1.003	0.244	4.122		0.000	0.000	2.038
N2b		1.535	1.159	2.033		1.982	1.332	2.948
N2c		1.661	0.954	2.894		2.393	1.197	4.782
N3		2.391	0.876	6.526		2.605	0.793	8.559
**Tumor location**	0.737				0.885			
Dorsal surface of tongue		1 (reference)				1 (reference)		
Border of tongue		0.949	0.581	1.552		1.225	0.498	3.015
Ventral surface of tongue		0.776	0.455	1.323		0.888	0.336	2.346
Anterior 2/3 of tongue		1.135	0.711	1.812		1.389	0.588	3.283
Overlapping lesion of tongue		0.643	0.358	1.154		1.200	0.457	3.153
Tongue anterior, NOS		0.936	0.588	1.488		1.037	0.442	2.431
**Surgery therapy**	0.000				0.000			
Yes		1 (reference)			0.000	1 (reference)		
No		2.856	2.267	3.599		3.687	2.561	5.308
**Grade**	0.000				0.013			
1		1 (reference)				1 (reference)		
2		1.184	0.941	1.491		1.256	0.871	1.812
3		1.607	1.212	2.132		1.713	1.085	2.703

*CI* confidence interval, *HR* hazard ratio, *NOS* not otherwise specified.

P values were calculated using an adjusted Cox proportional-hazards model.

### Subgroup analysis of the relationship between surgery and survival

Multivariate Cox regression analysis demonstrated that variables, including age, grade, T category, N category, and surgery therapies were all independent prognostic factors of OS and CSS. To rule out the effects of these variables and further validate the effect of surgery on OS and CSS, we conducted the subgroup analysis based on these variables (Table 3). Remarkably, the effect of no surgery was significantly associated with a higher risk of poor CSS in patients aged 75–79 years (HR 5.279; 95% CI 3.111 − 8.956; (P < 0.001)) and 80–84 years (HR 9.641; 95% CI 5.461–17.021; (P < 0.001)), and 85–102 years (HR 6.259; 95% CI 3.700–10.590; (P < 0.001)). Amongst those undergoing surgery, 16 (1.8%) patients died within 30 days after cancer diagnosis and 6 (0.7%), 3 (0.3%), and 7 (0.8%) patients were aged 75–79, 80–84, and 85–102 years old, respectively.

**Table 3:** The effect of surgery on overall survival and cancer-specific survival based on different subgroup variables.

Prognostic factor	Overall survival				Cancer-specific survival			
	P value	HR	Lower 95% CI	Higher 95% CI	P value	HR	Lower 95% CI	Higher 95% CI
**Gender**								
Male	0.000	4.198	3.142	5.610	0.000	6.194	3.924	9.777
Female	0.000	5.443	4.096	7.234	0.000	7.226	4.808	10.859
**Age**								
75–79	0.000	4.231	2.970	6.029	0.000	5.279	3.111	8.956
80–84	0.000	5.326	3.718	7.630	0.000	9.641	5.461	17.021
85–102	0.000	4.494	3.178	6.356	0.000	6.259	3.700	10.590
**Grade**								
1	0.000	9.844	6.391	15.163	0.000	15.357	7.883	29.918
2	0.000	4.879	3.728	6.385	0.000	6.143	4.108	9.186
3	0.005	1.954	1.225	3.116	0.003	2.850	1.414	5.745
**Race**								
Caucasian	0.000	4.975	3.983	6.212	0.000	6.553	4.681	9.175
Asian	0.000	4.470	2.206	9.058	0.001	5.644	1.960	16.251
African American	0.019	2.439	1.156	5.146	0.004	4.862	1.642	14.399
**Stage**								
I–II	0.000	6.569	4.719	9.144	0.000	18.902	10.801	33.080
III–IV	0.000	2.420	1.859	3.151	0.000	2.529	1.754	3.646
**T classification**								
T1	0.000	4.943	2.784	8.773	0.000	8.891	3.126	25.287
T2	0.000	3.744	2.741	5.116	0.000	6.425	3.965	10.410
T3	0.000	2.314	1.481	3.616	0.002	2.875	1.497	5.522
T4a	0.040	1.768	1.027	3.043	0.330	1.453	0.685	3.084
T4b	0.509	0.019	0.000	2359.993	–			
**N classification**								
N0	0.000	5.475	4.178	7.175	0.000	13.282	8.416	20.962
N1	0.000	3.059	1.883	4.967	0.010	2.453	1.244	4.836
N2a	0.616	434.450	0.000	> 1000	–			
N2b	0.007	1.961	1.199	3.209	0.098	1.698	0.907	3.178
N2c	0.173	2.190	0.709	6.768	0.120	3.636	0.715	18.487
N3	0.343	105.281	0.007	1,593,878.432	0.393	58.761	0.005	671,565.640
**Tumor location**								
Dorsal surface of tongue	0.007	3.960	1.456	10.772	0.121	6.936	0.599	80.262
Border of tongue	0.000	6.838	3.947	11.844	0.000	9.806	4.518	21.284
Ventral surface of tongue	0.000	4.307	2.164	8.571	0.000	9.476	2.851	31.492
Anterior 2/3 of tongue	0.000	5.642	3.863	8.239	0.000	6.304	3.429	11.588
Overlapping lesion of tongue	0.011	2.679	1.248	5.752	0.043	2.746	1.034	7.295
Tongue anterior, NOS	0.000	4.109	2.925	5.772	0.000	6.362	3.880	10.431

*CI* confidence interval, *HR* hazard ratio, *NOS* not otherwise specified.

P values were calculated using an adjusted Cox proportional-hazards model.

## Discussion

Surgery is rarely performed in elderly patients in clinical practice, considering that advanced age is associated with decreased functional status and increased co-morbidity, such as cardiovascular, respiratory, metabolic, hepatic, and renal diseases. Therefore, medical decision about whether elderly patients with OTSCC should undergo surgery or not should be made more carefully.

Surgery is widely believed to be the best way to treat solid tumors, and age shouldn’t be a decisive factor alone in medical decisions^16,17^. Several studies have reported that elderly patients with lung cancer^18^, papillary thyroid cancer^19^, colon cancer^20^, liver cancer^21^ and so on should still receive surgery after prognosis assessment and perioperative risk stratification. However, whether surgery should be performed on OTSCC patients aged ≥ 75 years old better was not further analyzed.

In our study, 1064 patients with OTSCC were included, but only 16.8% (179/1064) didn’t receive surgery to primary tumor, which indicated that the management of patients was in favor of surgery because more than 80% of the elderly patients had chosen surgery and the remaining 16.8% of patients might have too many risk factors to be deemed an appropriate surgical candidate. We also found that the rate of surgery declined with advancing age; however, subgroup analysis showed that survival advantage was associated with cancer-directed surgery at all age groups. Soudry et al. found that the 5-year disease-free survival (DFS) was 65% and 58% for patients over 75 and younger patients, and corresponding rates for 5-year disease-specific survival were 69% and 70%, which were not statistically significant. Patients with OTSCC aged 75 years or older should be managed like younger patients and they should be given a chance for treatment in terms of clinical staging and co-morbidities, because their prognosis is not different from that in younger patients^22^. What’s more, Mukdad et al. found that surgery predicted improved OS and DSS in all groups except young females (≤ 40 years)^8^. In addition, older age remained an independent risk factor for both OS and CSS even in such an elderly population, which has never been reported individually. Therefore, surgery could be prudently recommended to patients with optimistic life expectancies and carefully selected and closely observed OTSCC patients, including acceptable morbidity, anticipated life span, comorbidities, patient wishes, nutrition, functional status, and social support, could benefit from surgery. Better prognostic and predictive tools for select elderly patients for surgery are needed.

The greatest strength of this study is the population-based nature of the study and the large sample size, which gives insight to the nature of how US doctors treating elderly OTSCC patients. Several limitations of this study should be underlined. First, retrospective analysis may contribute to some bias. Second, due to the data limitations of the SEER database, the lack of data on additional predictors of OS such as comorbidities, performance status, and systemic therapy prevented us to adjust our analyses for these important factors.

## Conclusion

Our results showed that a series of factors contributed to poor outcomes in the elderly OTSCC patients, including clinicopathological characteristics and surgical management. Surgical resection is significantly associated with an improved prognosis, but further exploration in larger prospective clinical trials and better prognostic and predictive tools for select old patients for surgery are needed.

## Materials and methods

### Cohort population

The Surveillance, Epidemiology, and End Results (SEER) program registries collect data on patient demographics; cancer stage, site and type; treatment of the primary tumor; follow-up vital status; and OS and CSS. SEER*Stat Version 8.3.4 (http://www.seer.cancer.gov/seerstat) from the National Cancer Institute was used to identify eligible patients in this study. Tumor staging was assigned with corresponding criteria as described in the classification protocol developed by the American Joint Commission on Cancer (AJCC). Because the SEER database began collecting information on the presence or absence of metastases at the time of diagnosis in 2010, we included patients aged 75 years or older diagnosed with microscopically confirmed OTSCC between 1 January 2010 and 31 December 2014. We selected patients with only one primary malignancy in their lifetime^3^. A total of 1064 OTSCC patients were included.

### Statistical analysis

OS was calculated in months from the date of diagnosis to death, or the date of last follow-up and CSS time from the date of diagnosis to cancer-associated mortality or the date of last follow-up. Survival curves were generated by the Kaplan–Meier method. The Cox proportional hazard model was used to explore the effect of surgery on time to event. All statistical analyses were performed using SPSS (version 24.0) and GraphPad Prism (version 7.0). All reported P-values are two-sided with the level of significance set at 0.05.

### Ethical approval and informed consent

All procedures performed in studies involving human participants were in accordance with the ethical standards of Fudan University Shanghai Cancer Center Ethics committee and with the 1964 Helsinki declaration and its later amendments or comparable ethical standards. The experimental protocols were also approved by Fudan University Shanghai Cancer Center Ethics committee. Written informed consent was obtained from all individual participants included in the study.

## Data Availability

The datasets used and/or analysed during the current study are available from the corresponding author on reasonable request.
